# Examining the Association Between Occupational Strain and Risk of Angina Pectoris Among Older Working Adults in India Using Karasek’s Job Demand Control Model

**DOI:** 10.21203/rs.3.rs-8740583/v1

**Published:** 2026-02-19

**Authors:** Pravesh Kumar, Yoshiko Ishioka Miyata

**Affiliations:** O.P. Jindal Global University; O.P. Jindal Global University

**Keywords:** Angina Pectoris, JDC Model, Occupational Stress, Older adults, LASI, India

## Abstract

**Background:**

Occupational strain is a well-known predictor of cardiovascular diseases (CVDs), yet limited evidence exists for older workers in India. Using the Job Demand–Control (JDC) model, this study examines the association between job strain and the risk of Angina Pectoris (AP) among older workers, focusing on psychosocial workplace conditions linked to later life AP risk.

**Method:**

We used Wave 1 (2017–18) of the Longitudinal Aging Study in India (LASI), a nationwide survey, focusing on working older adults (*n* = 66,331). Occupational strain was conceptualised using the JDC model (high strain, active, passive, low strain), while AP was assessed using the Rose Angina Questionnaire. We employed a multivariable logistic regression model to examine the association between job strain and AP while adjusting for socioeconomic and health-related variables.

**Results:**

After adjusting for socioeconomic and health factors, individuals in both active (OR=0.77, 95% CI: 0.64–0.92) and passive jobs (OR=0.84, 95% CI: 0.74–0.96) exhibited a significantly lower likelihood of AP, whereas low-strain jobs showed a marginally protective but non-significant association compared to high-strain jobs. Early labour force participation (before age 14) and poorer self-rated health were associated with a higher risk of AP. Regional variation was significant, while socioeconomic variables were not significant after adjustment.

**Conclusion:**

This study highlights the role of psychosocial conditions, including occupational strain, in the development of angina in later life. The findings point to the need for better work environments that allow employees more control, decision-making autonomy, and greater skill discretion in their roles. At the same time, early identification and management of job stress and burnout may reduce the burden of angina and other cardiac events among India’s aging workforce.

## Introduction

Cardiovascular diseases (CVDs) remain a major cause of death worldwide. In 2022, an estimated 19.8 million people lost their lives due to CVDs, which was responsible for roughly one in three deaths. Nearly 85% of these deaths were due to heart attacks or strokes alone. What stands out is that more than 75% of CVD-related deaths occurred in low- and middle-income countries (LMICs). Of the nearly 18 million early deaths (before age 70) from noncommunicable diseases (NCDs) in 2021, close to 38% were linked to CVDs. The countries with the highest number of CVD deaths include China, India, Russia, the United States, and Indonesia [2].

In 2016, NCDs accounted for 63% of all deaths in India, with CVDs alone resulting in 27% deaths [3]. The age-standardised CVD mortality rate in the same year stood at 282 deaths per 100,000 population, which was higher than the global average of 233 per 100,000, highlighting a serious public health issue [4].

Given the serious socio-economic consequences of these trends, India set a target of reducing premature mortality due to CVDs and other major NCDs by 25% by 2025 under the National Health Policy, 2017 [5]. However, achieving this target remains a major challenge given the estimated economic loss of US$4.58 trillion due to NCDs by 2030, of which nearly half (US$2.17 trillion) is attributed to CVDs alone [6].

One primary clinical manifestation of this burden is Angina Pectoris (AP), a condition characterised by chest pain or discomfort caused by coronary heart disease. AP occurs when the blood supply to the heart muscle does not meet its demand due to obstruction or spasm of the coronary arteries [7]. As angina precedes serious cardiac events, identifying and addressing its risk factors, including psychosocial stressors such as job strain, is critical for reducing the burden of CVDs and associated deaths in India.

Studies across the globe have shown mixed results about the association between an increase in the risk of CVDs and stress at work, which has been measured by the Demand-Control Model (DCM) [8–10]. Despite this variability, the association between this model and CHD has been found to be strong and consistent [12–14].

Several studies have demonstrated that high job strain, characterised by high psychological demands combined with low decision control, is associated with increased cardiovascular risk. For instance, Schnall et al. (1994) observed that job strain (high psychological demands and low decision control) was associated with CVDs, especially AP [14]. Similarly, a Canadian community-based study established that job strain was associated with an increased risk of heart disease in older adults [15]. Likewise, a prospective cohort study further demonstrated that employees in high-strain jobs had twice the CVD mortality risk compared to their counterparts in low-strain jobs [16]. Karasek et al. (1981) also reported that higher job demands and low intellectual discretion are significant predictors of the development of CHD [17]. In addition, one study highlighted exogenous factors like unfavourable occupational conditions, excessive workload, lack of teamwork and endogenous pressures such as individual personality characteristics, are potential risk factors for acute coronary syndromes [18].

Several large scale studies have found an association between high job strain and risk of developing cardiovascular conditions, including the Copenhagen Psychosocial Questionnaire (COPSOQ-1) study, INTERHEART case-control Study, NHANES1 study, Danish Work Environment Cohort Study (DWECS), Finnish Public Sector (FPS) study, WOLF Stockholm study, Intervention Project on Absence and Well-being (IPAW) study, Northern Italian Study, Women’s Health Study, and Mid-life in the United States (MIDUS) cohort study [19–28].

On the contrary, other major studies have reported that job strain was not correlated with cardiovascular conditions among workers. These include the Framingham Offspring Study, Nurses’ Health Study, and WHI-OS (Women’s Health Initiative Observational Study), European cohort studies, Swedish Primary Prevention Study (PPS), Swedish Västerbotten Intervention Programme, and Belgian Job Stress Project (BELSTRESS) [29–35]. A similar association was documented in previous studies [36–38].

In the Indian context, several studies have identified low-density lipoprotein-cholesterol (LDL-C) [39], type-2 diabetes mellitus (T2DM) [40,41], obesity [42,43], smoking [44], insomnia or sleep disorder [45,46], hypertension [47,48], family history of CVD [49,50] and physical inactivity or sedentary lifestyle [51] as major modifiable risk factors for CVDs.

However, the cause of AP due to occupational risk factors is not fully understood in India, particularly among the older working population. To address this gap, the present study examines the association between job strain and AP using nationally representative data. To our knowledge, this is the first study in India to use two globally validated instruments: the Rose Angina Questionnaire (RAQ) for assessing AP and Karasek’s Job Demand-Control (JDC) model for measuring job strain. In addition, our study includes several understudied occupational variables, such as age at entry into the workforce and intention to leave current occupation, among others that have rarely been explored in previous studies. This combination of validated tools, representative data, and unique occupational variables adds novel evidence on the relationship between job strain and AP in India, with important implications for both research and policy.

### Karasek’s Job Demand-Control (JDC) Model

The JDC model, introduced by Robert Karasek in 1979, uses 18-item scale of the Job Content Questionnaire (JCQ) to combine psychological demands at work with job decision latitude or control in four categories, or quadrants: 1) low strain (low psychological demands and high decision latitude); 2) passive (low psychological demands and low decision latitude); 3) active (high psychological demands and high decision latitude); and 4) high strain (high psychological demands and low decision latitude) [52,53].

Psychological demands refer to the mentally demanding aspects of a main job, such as working under time pressure, heavy workload, strenuous tasks, and job complexity. In contrast, job decision latitude, also known as job control, refers to the level of autonomy and skill discretion an employee has in performing their work or assigned activities [54–56]. The high strain quadrant is related to the highest risk of adverse health, and the low strain quadrant is proposed to have the lowest risk, whereas the active and passive quadrants have intermediate risks [54].

Based on the above, this study examines the association between job strain and AP using nationally representative data from India. We hypothesize that the risk of AP is higher among individuals employed in high-strain jobs compared to those in other jobs.

## Method

### Study Design and Setting

We used Wave 1 of the Longitudinal Aging Study in India (LASI), a nationally representative study in India. LASI covers 73,396 individuals aged 45 and above, including their spouses, regardless of age. The sample included 31,902 older adults (aged 60 and above) and 6,880 oldest-old individuals (aged 75 and above) drawn from all states and union territories of India. Data collection for LASI Wave 1 took place between April 2017 and December 2018.

Households in LASI were considered eligible if they included at least one member aged 45 years or older. LASI offers detailed information on the socio-economic and health dimensions of the aging population in India and is harmonized with the HRS in the United States and other aging surveys in the world, for instance, CHARLS, SHARE, MHAS, KLoSA and JSTAR. The study employed a multistage, stratified, area-probability cluster sampling design to arrive at the final sample [57,58].

All demographic, occupational, and health-related variables used in this study were derived and recoded from items included in the survey instrument, which is available for research purposes and provides a detailed description of items and response categories relevant to each construct (lasiindia.org/public/documentation/LASI_Questionnaire.pdf#page = 182.05). Moreover, this study is a secondary analysis and does not involve any intervention; therefore, clinical trial registration is not applicable.

### Outcome variable

We assessed the presence of AP using the Rose Angina Questionnaire (RAQ) from the World Health Organisation, which is a validated instrument for detecting angina in population-based studies [59]. Respondents were confirmed to have angina if they ever experienced exertional chest pain or discomfort that met the following criteria: 1) pain occurring during physical activity such as “walking uphill, hurrying, or walking at a normal pace on level ground”; 2) pain located in the “sternal region or the left side of the chest, with or without radiation to the left arm”; 3) pain severe enough to “cause the individual to stop or slow down”; and 4) symptoms that “resolved within 10 minutes after stopping or reducing activity.” We summed these four cardiovascular conditions and coded them into a binary variable (0 = No, 1 = Yes) [60].

### Explanatory variables

Further, to account for the strain in the main job/occupation we used an adapted Job Content Questionnaire (JCQ), which is based on the Job Demand-Control (JDC) model by combining nine occupational demand items available in the original survey, wherein respondents were asked how often their job required the following tasks: 1) “a lot of physical effort”, 2) “lifting heavy loads”, 3) “stooping, kneeling, or crouching”, 4) “good eyesight”, 5) “intense concentration or attention”, 6) “skill in dealing with other people”, 7) “exposure to burning material, exhaust, or smoke (excluding car exhaust)”, 8) “exposure to chemicals, pesticides, or herbicides”, and 9) “exposure to noxious odors” [58]. These demand items were averaged, reverse-coded (1 = low, 4 = high) and then categorised into four quadrants: 1) “High Strain (high psychological demand, low control)”, 2) “Active (high demand, high control)”, 3) “Passive (low demand, low control)”, and 4) “Low Strain (low demand, high control) [61]. These items had acceptable internal reliability, with a Cronbach’s α of 0.71.

## Covariates

### Demographic and social background

Age was categorised into < 45 years, 45–59 years, and ≥ 60 years. Sex was coded as male or female. Marital status was classified as currently married, widowed, or not in marital union. Educational attainment was grouped into no education, primary, secondary, and higher education. Caste was categorised as Scheduled Caste, Scheduled Tribe, Other Backwards Classes, or Others. Religion was classified as Hindu, Muslim, Christian, or Other. Place of residence was coded as rural or urban, while geographic region was classified into six major regions based on state of residence.

### Socioeconomic position

Household economic status was measured using an abridged version of the NSS consumption expenditure schedule, which collected information on 11 food and 29 non-food expenditure items on the basis of reference periods of 7 days and 30 days preceding the survey. This information was used to compute the “monthly per capita consumption expenditure (MPCE)”, which in turn represents overall household consumption levels. In the original survey, MPCE was categorised into five quintiles ranging from the poorest to the richest, which we subsequently regrouped into three categories in our analysis: “Poor (lowest two quintiles)”, “Middle (third quintile)”, and “Rich (top two quintiles)” [62].

### Lifestyle behaviors

Smoking history was defined as a binary variable based on a history of smoking (cigarette, bidi, cigar, hookah, cheroot) or smokeless tobacco (chewing tobacco, gutka, pan masala, etc) products [63]. Alcohol use was similarly coded into a dichotomous variable based on lifetime consumption of both commercially and traditional or locally produced alcoholic beverages.

### Occupational and work-history characteristics

In relation to the legal working age of 14 in India, age at first work was categorised as early (< 14 years) and late (≥ 14 years)

Further, the work status (0 = employed, 1 = self-employed) of individuals was assessed based on responses to two survey items. Respondents were classified as “self-employed” if they reported owning a business or farm or identified their occupation as private or entrepreneur. They were classified as “employed” if they reported working in government, public sector, private sector (non-entrepreneur), as a casual labourer, or in other employment types without owning a business or farm. Non-workers or those with incomplete data were coded as missing in our study. Further, respondents in seasonal or temporary jobs/occupations were also dropped from our study.

In addition, job turnover intention was generated as a binary variable (0 = Intention to Stay, 1 = Intention to Leave) based on responses to four survey items asked of respondents who reported ever working. Respondents were asked whether: 1) they were currently looking for another job, 2) they registered with an employment exchange, 3) they registered with the MGNREGA, which was asked only in rural areas, and 4) they had contacted prospective employers in the past month. The variable was categorised into “Intention to Leave (ITL)” if respondents answered yes to any of these questions, which indicates active job-seeking behaviour and “Intention to Stay (ITS)” if respondents gave no positive responses to these three questions. Non-workers and respondents with incomplete work histories were excluded from the present study.

### Functional health and disability

We assessed difficulties in performing activities of daily living (ADL) and instrumental activities of daily living (IADL) because of physical, mental or cognitive issues using the Barthel Index (BI) [64]. For ADL, respondents were asked about difficulties faced by them in performing six daily activities: 1) “dressing, including putting on footwear”, 2) “walking across a room”, 3) “bathing”, 4) “eating”, 5) “getting in or out of bed”, and 6) “using toilet including sitting down and standing up”. For IADL, respondents were asked about difficulties in seven important activities: 1) “preparing a hot meal including cooking and serving”, 2) “grocery shopping”, 3) “making telephone calls”, 4) “taking prescribed medicines”, 5) “doing household or garden work”, 6) “managing finances such as paying bills or tracking expenses”, and 7) “moving around or navigating unfamiliar places”. Each item was coded as binary (0 = No difficulty, 1 = Difficulty) based on responses of “Yes” or “No”, excluding difficulties expected to last less than three months. An ADL score (0–6) was calculated by summing the six ADL items, and an IADL score (0–7) was calculated by summing the seven IADL items. We created three categories for the ADL score: “No” (score = 0), “Moderate” (score = 1–2), and “Severe” (score ≥ 3) difficulty. Similarly, the IADL score was categorised into three levels: “No” (score = 0), “Moderate” (score = 1–2), and “Severe” (score ≥ 3) difficulty.

### Subjective health and well-being measures

We classified self-rated health (SRH) into three levels: Poor (coded as 0), Moderate (coded as 1), and Good (coded as 2) by merging “good” and “fair” into a single category. Further, we generated satisfaction with life using the “Satisfaction with Life Scale (SWLS)”, which measures overall quality of life. Respondents in the survey rated their life based on five evaluative statements: 1) “my life is close to ideal”, 2) “the conditions of my life are excellent”, (3) “I am satisfied with my life”, (4) “I have achieved the important things I want in life”, and (5) “I would change almost nothing if I could live my life over”. Each item was recorded on a seven-point Likert scale ranging from Strongly Disagree (1) to Strongly Agree (7). A total life satisfaction score was calculated by summing the responses to these five items, ranging from 5 to 35. The score was then categorised as “Low” (5–20), “Medium” (21–25), or “High” (26–35) [65]. These items demonstrated excellent internal reliability, with a Cronbach’s α of 0.89.

### Mental health and sleep indicators

We evaluated the presence of depressive symptoms using a 10-item version of the “Centre for Epidemiologic Studies Depression (CES-D) Scale”. We generated a binary variable (0 = Not depressed, 1 = Depressed) based on a set of questions from LASI survey about symptoms experienced by respondents over a period of two weeks or more in a year. Respondents were asked whether they: 1) “felt depressed”, 2) “found everything to be an effort”, 3) “had sleep restlessness”, 4) “lost interest in most things”, 5) “felt unusually tired or lacking energy”, 6) “experienced loss of appetite”, 7) “had increased appetite” (if no loss of appetite was reported), 8) “faced greater difficulty concentrating”, 9) “felt worthless or down on themselves”, and 10) “thought a lot about death (their own, others’, or in general)”. Responses to feeling depressed, sleep restlessness and everything being an effort were based on frequency, ranging from every day, almost every day, less often, with respondents reporting these symptoms every day or almost every day being said to have depressive symptoms. Other symptoms were binary (Yes/No). We calculated a CES-D score (0–10) by summing the presence of these symptoms, with a score of 4 or higher indicating the presence of depression [66,67].

The presence of insomnia symptoms (coded as 0 for ‘No’ and 1 for ‘Yes’) was assessed using the Jenkins Sleep Scale (JSS-4), which remains a widely used scale for evaluating sleep disorders in population-based studies. In the LASI survey, respondents were asked four questions about sleep difficulties experienced over the past month, which included how often they had 1) “difficulty falling asleep”, 2) “waking up at night and struggling to return to sleep”, 3) “waking up too early and being unable to fall asleep again”, and 4) “feeling unrested during the day despite adequate sleep”. Each item was rated as “Never or rarely” (1–2 nights per week), “Occasionally” (3–4 nights per week), or “Frequently” (5 or more nights per week). Following Jenkins et al. (1988), respondents were classified as having insomnia if they reported experiencing any of these symptoms occasionally or frequently [68].

### Clinical morbidity and subjective health complaints

Morbidity status was assessed using self-reported diagnoses of nine specific conditions: 1) hypertension or high blood pressure, 2) diabetes or high blood sugar, 3) cancer or a malignant tumor, 4) chronic lung disease such as asthma, chronic obstructive pulmonary disease, chronic bronchitis, or other chronic lung problems, 5) chronic heart disease such as coronary heart disease, congestive heart failure, or other chronic heart problems, 6) stroke, 7) musculoskeletal conditions such as arthritis, rheumatism, osteoporosis, or other disorders of the bones and joints, 8) neurological and mental health conditions including depression, Alzheimer’s disease, dementia, bipolar/unipolar disorders, seizures, or Parkinson’s disease, and 9) elevated cholesterol levels [69]. We recoded each condition as “Yes” or “No” based on self-reported conditions. We then defined the morbidity status as “no morbidity” (coded as 0) if respondents reported no conditions, “single morbidity” (coded as 1) if one condition was reported, and “multimorbidity” (coded as 2) if two or more conditions were reported.

Finally, the presence of subjective health complaints (SHC) captured on the basis of self-reported symptoms experienced in the past two years: 1) “pain/stiffness in joints”, 2) “persistent swelling of lower limbs”, 3) “breathlessness while awake”, 4) “persistent dizziness or light headedness”, 5) “back pain”, 6) “persistent headaches”, 7) “severe fatigue or exhaustion”, 8) “wheezing/whistling sound from the chest”, and 9) “cough with or without sputum”. If respondents reported any of these symptoms, they were classified into “Yes” (coded as 1) and “No” (coded as 0) if they reported otherwise [70].

### Statistical analysis

We analysed the data in Stata version 17 (StataCorp, 2017. Stata Statistical Software: Release 17. College Station, TX: StataCorp LP).

Before we began statistical analysis, we checked for potential multicollinearity for all explanatory variables using the Variance Inflation Factor (VIF). The mean VIF was 1.56, and all individual VIFs were well below the conventional threshold of 10 (and even the conservative threshold of 5), which indicates no serious multicollinearity problem in our model. We then executed the *svyset* command to account for the complexity of the survey design of LASI and to adjust for sample weight in our analysis. We used the chi-square (χ^2^) test to examine the association between AP and each predictor variable under study and obtained p-values from this analysis. We employed a binary logistic regression model in our study to understand the association between the outcome and predictor variables. The equation for the logistic regression model can be defined as follows:

$$\text{log}\left(\frac{pi}{1-pi}\right)=\text{logit}\left(pi\right)=\beta_{0}+\beta_{x1}+\beta_{x2}+\ldots \;\beta_{n}x_{n}$$

In the equation given above, *pi* denotes the probability of an individual *i* experiencing AP, while 1 − *pi* is the probability of an individual *i* not experiencing AP. *x*1, *x*2..*Xn* are the predictors, *β*_0_ is the intercept and *β*_1_, *β*_2_….*β*_n_ are the coefficients. The coefficients have been exponentiated to obtain odds ratios (OR), which represent the change in the odds of AP associated with a one-unit change in the predictor, while holding other factors constant.

We used two models in our study. In the first model, we used a univariate logistic regression model to derive crude odds ratios (ORs) to assess the independent effect of occupational strain on AP without adjusting for confounders. In the second model, we fitted a multivariable logistic regression model by including all covariates. This allowed us to estimate the adjusted odds ratios (AORs) while accounting for potential confounders. We obtained p-values based on logistic regression analysis. *p* < 0.05 is said to be statistically significant, while *p* < 0.001 is highly significant.

We also estimated the predicted probabilities of AP in relation to occupational strain by giving the *margins* command, and visualised these probabilities by applying the *marginsplot* command.

### Ethical Considerations

In accordance with ethical guidelines, written informed consent was obtained from all age-eligible participants by the LASI survey team. The study protocol was reviewed and approved by the Institutional Review Boards of the Indian Council of Medical Research (ICMR, F.No.T.21012/07/2012-NCD), the International Institute for Population Sciences (Sr. No. 12/1054), the Harvard T.H. Chan School of Public Health (CR-16715–10), and the University of Southern California (UP-CG-14_00005) [70,71].

## Results

### Sample Characteristics and Regional Variation in the Prevalence of AP

Table 1 shows the prevalence of AP among older adults in India. Overall, about 11% (approximately 12 out of every 100) reported angina symptoms. Prevalence varied modestly by region and job quadrant. Regionally, AP was highest in the central region (17.65%), followed by the western region (12.86% and lowest in the south (9.68%) and northeast (9.75%). By job quadrants, the highest prevalence was seen among older adults in active jobs (12.02%), followed by high-strain (11.33%), low-strain (11.27%), and passive jobs (10.85%).

### Sociodemographic Predictors of AP among Older Adults in India

Table 2 summarises findings from the logistic regression model, which shows the association between AP and various sociodemographic, health, and work-related characteristics among older adults in India.

Compared with those under 45 years, individuals aged 45–59 years were 19% less likely to have AP (AOR = 0.81; 95% CI: 0.68–0.97), and those aged 60 years or older were 27% less likely (AOR = 0.73; 95% CI: 0.60–0.90). Women were 26% more likely to have angina than men (AOR = 1.26; 95% CI: 1.11–1.44).

Relative to currently married respondents, those who were divorced, separated, deserted, or in other marital statuses were 29% less likely to have angina (AOR = 0.71; 95% CI: 0.53–0.93), while we found no association for widowed individuals.

Education showed a protective trend: secondary education was 17% less likely to have angina (AOR = 0.83; 95% CI: 0.71–0.96), and higher education was 37% less likely (AOR = 0.63; 95% CI: 0.51–0.78) compared with no education. We found no association for primary education. We also found no association between caste, religion, or socioeconomic status (MPCE) and AP after adjustment. Those living in urban areas had a 32% lower likelihood of angina compared with those residing in rural areas (AOR = 0.68; 95% CI: 0.61–0.76).

Regional differences were observed relative to the North. The East was 22% less likely to have AP (AOR = 0.78; 95% CI: 0.66–0.92), while the West was 43% more likely (AOR = 1.43; 95% CI: 1.21–1.69), and the Central region was 114% more likely (AOR = 2.14; 95% CI: 1.77–2.58). The South or Northeast regions were not significant.

Entering the workforce at or after age 14 was 18% less likely to have AP than entering before age 14 (AOR = 0.82; 95% CI: 0.73–0.93). The current employment status (self-employed vs. employed) was not associated with AP.

Compared with high-strain jobs, passive jobs (AOR = 0.84; 95% CI: 0.74–0.96) were 16% less likely to have AP, and active jobs were 23% less likely (AOR = 0.77; 95% CI: 0.64–0.92). On the other hand, low-strain jobs (AOR = 0.89; 95% CI: 0.80–1.01, *p* = 0.063) were not statistically significant but showed a marginally protective effect.

**Figure 1:**
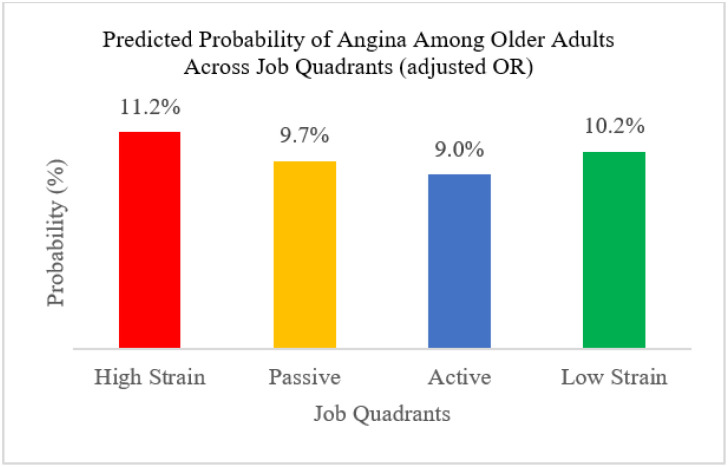
Predicted Probability of AP across Job Quadrants

In accordance with the JDC model, predicted probabilities were highest in high-strain jobs and lowest in active jobs (Fig. 1), supporting the role of high job demands combined with low control in negative health outcomes.

Those with a higher intention to leave (ITL) their current occupation had 45% higher adjusted odds of angina than those without ITL (AOR = 1.45; 95% CI: 1.18–1.79).

For functional limitations, moderate ADL were 35% more likely to report AP than no limitations (AOR = 1.35; 95% CI: 1.15–1.58), while severe limitations were not significant. In contrast, moderate IADL limitations were 20% more likely (AOR = 1.20; 95% CI: 1.06–1.36), and severe IADL limitations were 39% more likely (AOR = 1.39; 95% CI: 1.18–1.63) compared to no IADL limitations.

A smoking history was 23% more likely to have AP (AOR = 1.23; 95% CI: 1.11–1.37), whereas alcohol consumption was not associated.

Moderate SRH was 25% less likely to experience AP (AOR = 0.75; 95% CI: 0.66–0.85), while good SRH was 58% less likely (AOR = 0.42; 95% CI: 0.37–0.49) compared to poor SRH. Medium life satisfaction was 17% more likely to report AP (AOR = 1.17; 95% CI: 1.03–1.32). We found no association with high satisfaction. Depressive symptoms had 28% more likelihood of AP (AOR = 1.28; 95% CI: 1.09–1.51), and insomnia symptoms were 93% more likely to have AP (AOR = 1.93; 95% CI: 1.73–2.15). Subjective health complaints in the past two years were also 104% more likely to have AP (AOR = 2.04; 95% CI: 1.13–3.68).

Finally, while individuals with single morbidity reported 59% higher likelihood of having AP (AOR = 1.59; 95% CI: 1.42–1.78), and those with multiple morbidities were 131% more likely (AOR = 2.31; 95% CI: 2.02–2.65) compared to those with no morbidity.

## Discussion

Our study examined the relationship between occupational strain, psychosocial factors, behavioural risks, multimorbidity, and AP among working older adults in India using Karasek’s JDC model. We noted several key findings in our study.

First, those who entered the workforce early (before the age of 14) reported a higher likelihood of AP. Second, high job strain was linked to a higher risk of AP, whereas those in active jobs showed the lowest risk, as confirmed by predicted probabilities. Third, psychosocial factors such as poor self-rated health, depression, insomnia and subjective health complaints were associated with elevated risks of angina in our study. Fourth, limitations in IADL and ADL and single or multiple morbidities substantially raised the risk of AP. Fifth, rural residents reported a higher risk in comparison to urban residents. Sixth, subjective health problems stood out as a strong predictor of angina in our analysis. Finally, a clear regional variation emerged as a strong predictor of angina after adjustment.

We observed that the risk of AP was less common among older age groups (45–59 years and ≥ 60 years) compared with individuals younger than 45 years. Although the risk of CVDs typically increases with age, this pattern may be due to the characteristics of our working sample, where older adults who remain in the labour force tend to be healthier than those who have already withdrawn due to illness [72,73]. Second, older adults may experience underdiagnosis or under-reporting of angina [74], lower health-seeking behaviour [75], or normalisation or underestimation of symptoms as part of aging, particularly in resource-constrained settings [76].

Further, elderly women in our study were more likely to report angina than elderly men, a pattern widely reported in studies from both high- and middle-income countries [77–79]. Older adults who were not in a marital union were less likely to have angina than those who were currently married, which is in line with an earlier study that the prevalence of AP was higher among those in a marital union [80]. We found an inverse relationship between educational level and AP, where individuals with lower educational attainment exhibited a higher risk of CHD [81–84]. Interestingly, no significant association was observed between caste, religion, or MPCE and risk of AP after adjusting for key covariates. We assume that in large population surveys, these factors may not always be strong predictors of self-reported medical conditions, including angina, due to underreporting and underdiagnosis of CVDs in a country like India [85,86]. Older adults living in urban areas were less likely to experience angina than their rural counterparts, which is in agreement with national and international estimates [57,87–89]. Likewise, we observed a large state-level variation in angina burden in our study, which has been documented in various studies in the past [90–92]. These variations may be attributed to factors such as lower awareness, poor treatment-seeking behaviour and the presence of higher undiagnosed cases in rural settings.

Our results reaffirm that smoking is a major risk factor for AP, consistent with a large body of literature from across the globe [93–95]. We noted no association between drinking alcohol and elevated risk of AP. Studies have shown mixed results. For instance, Merry et al. (2011) found no clinically significant evidence to support this relationship [96], while others reported a meaningful association [97,98].

It was further noted that early initiation of the work significantly raises the risk of AP, which conceptually aligns with findings from previous studies [99]. In contrast, our analysis found no association between employment status and the risk of angina. We believe that the nature of employment alone may not capture the conditions that can increase the risk of AP [100]. We, therefore, recommend that future studies consider physical work environment, such as a noisy workplace, long working hours, variation in shift and physical demands, rather than employment status per se, as the more proximate determinants of CVD, including AP.

Furthermore, adjusted odds ratios revealed an unexpected pattern for passive and active jobs, which should be interpreted with caution in light of mixed evidence across studies. [27,55,101–104]. However, predicted probabilities derived from the same model revealed a pattern consistent with the JDC model, where the highest probability of angina was observed in high-strain and passive jobs. These roles are known to activate the stress response systems, e.g. HPA axis and sympathetic nervous system in the body [105,106], which trigger stress hormones and result in increased cardiovascular activity, including promotion of arterial inflammation [107,108]. Over time, repeated activation of these stress systems creates an accumulated physiological burden, referred to as ‘allostatic load’, which speeds up the buildup of plaque in the arteries and reduces cardiac blood flow. This in turn raises the risk of angina and other cardiovascular events [109]. In contrast, these probabilities were lowest in active and low-strain jobs due to lower allostatic load in the body and higher skill discretion and autonomy in jobs.

Further, in line with Sara et al. (2018) and Kachi et al. (2019), we noted that those with a higher turnover intention, possibly due to higher job dissatisfaction or burnout, were more likely to experience adverse health outcomes, including AP[110,111].

Likewise, older adults with functional limitations, especially limitations in performing IADL, were at higher risk of AP. This pattern is consistent with earlier studies10[112–114]. We also found an inverse association between self-rated health and angina, consistent with existing studies [115–117]. Accordingly, psychological distress was significantly associated with AP in our study, supported by studies across countries [11,73,100].

Moreover, older adults who exhibited insomnia symptoms were at higher risk of AP than those without these symptoms, which is in tune with studies that poor sleep and/or self-reported sleep disturbances are positively linked to CVD, including stable angina pectoris (SAP) and unstable angina pectoris (UAP) [118–121]. In accordance with previous literature, we observed that those with single or multiple morbidities were more likely to develop AP in our study [94,122,123]. Our findings further suggest that older adults who reported subjective health complaints over the last two years were more likely to experience AP, a finding consistent with prior studies [116–118].

In summary, our study contributes to a growing body of literature that behavioural and non-behavioural factors determine the risk of angina. In our study, geography emerged as a strong predictor of risk. These regional disparities are likely due to differences in access to healthcare, health-seeking behaviour, lifestyle factors, dietary patterns, socioeconomic and cultural background and awareness related to screening and diagnoses of AP, which highlights the need to introduce locally responsive public health interventions and target key determinants of CVDs. The absence of associations between caste, religion, and SES suggests that the risk of AP may be distributed across social groups in ways that may not be fully captured by traditional measures. By and large, our results resonate with the propositions outlined in the JDC that individuals exposed to high-strain jobs experience elevated risks of AP. In addition, subjective health problems emerged as an important predictor of angina in our study [124–126].

In conclusion, our study calls for improvements to existing laws in India, such as the NPSHEW, 2009, Factories Act, 1948, and OSH&WC Code, 2020. We also recommend early management of job-related stress and burnout, providing workplace counselling and improving the working conditions of female workers.

### Strengths and limitations of the study

A major strength of this study lies in the use of a large and nationally representative sample, supporting the generalizability of the findings. To our knowledge, this is among the first studies in India to utilised the globally validated JDC model to examine AP risk among older adults. However, this study has some inherent limitations due to its cross-sectional design and the pending release of wave 2 data, which restricts our ability to establish a causal association. Furthermore, we cannot exclude the possibility of recall bias, particularly in self-reported variables such as MPCE, self-rated health and chronic morbidities. Finally, unmeasured confounders in this study, such as family history of heart disease, hypertension, BMI, physical inactivity and other work-related variables, can be considered in future analysis.

The LASI project was co-funded by the NIA/NIH (R01 AG042778), the Ministry of Health & Family Welfare, the Government of India, and the United Nations Population Fund India. It was developed and collected by the International Institute for Population Sciences (IIPS), Harvard T. H. Chan School of Public Health (HSPH), & University of Southern California (USC). The authors declare that they were not involved in the design of the study or the collection of data, and that they received no funding to do this research.

## Figures and Tables

**Table 1:** Background Characteristics of Older Adults with Angina Pectoris (AP) (*n* = 66,331), LASI Wave-1 (2017–18)

Characteristics	Absence of AP		Presence of AP		
	(N = 58,616)		(N = 7,715)		
	n	%	n	%	p-value
**Age (years)**					
<45	6,129	90.44	648	9.56	< 0.001
45–59	30,834	89.03	3,801	10.97	
60+	21,653	86.89	3,266	13.11	
**Sex**					
Male	24,993	90.09	2,749	9.91	< 0.001
Female	33,623	87.13	4,966	12.87	
**Marital Status**					
Currently in marital union	47,223	88.74	5,990	11.26	< 0.001
Widowed	9,419	85.91	1,545	14.09	
Divorced/Separated/Deserted/Others	1,971	91.63	180	8.37	
**Level of Education**					
No education	25,297	86	4,118	14.00	< 0.001
Primary	14,377	87.62	2,031	12.38	
Secondary	12,280	91.38	1,159	8.62	
Higher	6,662	94.24	407	5.76	
**Caste**					
SC	9,681	87.32	1,406	12.68	< 0.001
ST	10,546	89.95	1,178	10.05	
OBC	22,154	88.04	3,010	11.96	
Others	14,127	88.52	1,832	11.48	
**Religion**					
Hindu	42,779	88.08	5,789	11.92	< 0.001
Muslim	6,890	87.16	1,015	12.84	
Christian	5,931	91.02	585	8.98	
Others	3,011	90.23	326	9.77	
**MPCE**					
Rich	23,407	87.58	3,320	12.42	< 0.001
Middle	11,800	88.66	1,510	11.34	
Poor	23,409	89.03	2,885	10.97	
**Place of Residence**					
Rural	36,969	86.63	5,705	13.37	< 0.001
Urban	21,647	91.5	2,010	8.5	
**Region**					
North	12,834	88.01	1,749	11.99	< 0.001
South	14,232	90.32	1,526	9.68	
East	10,320	89.3	1,236	10.70	
West	7,837	87.14	1,157	12.86	
Central	3,701	82.35	793	17.65	
Northeast	8,776	90.25	948	9.75	
**Smoking**					
No	38,669	88.93	4,812	11.07	< 0.001
Yes	19,393	87.12	2,867	12.88	
**Drinking**					
No	48,328	88.29	6,411	11.71	0.614
Yes	9,748	88.46	1,272	11.54	
**Age at first job**					
Before 14	6,039	83.75	1,172	16.25	< 0.001
At or after 14	52,577	88.93	6,543	11.07	
**Employment Status**					
Employed	4,970	24.70	466	19.89	< 0.001
Self-employed	15,152	75.30	1,877	80.11	
**Current Main Job Quadrants**					
High Strain	11,791	88.67	1,506	11.33	0.007
Passive	6,957	89.15	847	10.85	
Active	30,982	87.98	4,233	12.02	
Low Strain	8,886	88.73	1,129	11.27	
**Job Turnover Intention**					
Intention to Stay	28,635	88.99	3,541	11.01	< 0.001
Intention to leave	1,024	85.05	180	14.95	
**ADL Limitation**					
No difficulty	52,294	89.46	6,162	10.54	< 0.001
Moderate	4,460	80.32	1,093	19.68	
Severe	1,862	80.19	460	19.81	
**IADL Limitation**					
No difficulty	42,556	90.45	4,492	9.55	< 0.001
Moderate	9,157	84.77	1,645	15.23	
Severe	6,903	81.39	1,578	18.61	
**Self-Rated Health (SRH)**					
Poor	7,386	78.25	2,053	21.75	< 0.001
Moderate	23,067	86.44	3,619	13.56	
Good	27,618	93.23	2,004	6.77	
**Life Satisfaction**					
Low	16,981	87.52	2,421	12.48	< 0.001
Medium	14,300	87.86	1,975	12.14	
High	26,005	89.08	3,187	10.92	
**Depressive Symptoms**					
No	54,527	89.11	6,665	10.89	< 0.001
Yes	3,191	76.49	981	23.51	
**Insomnia**					
No	49,279	90.61	5,108	9.39	< 0.001
Yes	9,121	77.78	2,605	22.22	
**Morbidity Status**					
No morbidity	34,388	91.67	3,124	8.33	< 0.001
Single morbidity	15,129	86.16	2,431	13.84	
Multimorbidity	9,099	80.82	2,160	19.18	
**Subjective Health Complaints (SHC)**					
No	862	94.41	51	5.59	< 0.001
Yes	57,306	88.22	7,653	11.78	

Notes: Reported p-values are from Pearson’s chi-square tests, where smaller p-values indicatestronger evidence of an association between the variables

**Table 2:** Univariate and multivariable logistic regression of Angina Pectoris (AP)

Characteristics	Univariate		Multivariable	
	Unadjusted OR [95% CI]	P-value	Adjusted OR [95% CI]	P-value
**Age (years)**				
<45	ref			
45–59	1.17 [1.07–1.27]	0.001	0.81 [0.68–0.97]	0.02
60+	1.43 [1.31–1.56]	< 0.001	0.73 [0.60–0.90]	0.002
**Sex**				
Male	ref			
Female	1.34 [1.28–1.41]	< 0.001	1.26 [1.11–1.44]	< 0.001
**Marital Status**				
Currently married	ref			
Widowed	1.29 [1.22–1.37]	< 0.001	0.98 [0.85–1.13]	0.819
Divorced/Separated/Deserted/Others	0.72 [0.62–0.84]	< 0.001	0.71 [0.53–0.93]	0.015
**Level of Education**				
No education	ref			
Primary	0.87 [0.82–0.92]	< 0.001	0.91 [0.80–1.03]	0.137
Secondary	0.58 [0.54–0.62]	< 0.001	0.83 [0.71–0.96]	0.014
Higher	0.38 [0.34–0.42]	< 0.001	0.63 [0.51–0.78]	< 0.001
**Caste**				
SC	ref			
ST	0.77 [0.71–0.83]	< 0.001	0.88 [0.74–1.04]	0.138
OBC	0.94 [0.87–1.00]	0.053	0.96 [0.85–1.09]	0.553
Others	0.89 [0.83–0.96]	0.003	0.95 [0.82–1.12]	0.564
**Religion**				
Hindu	ref			
Muslim	1.09 [1.01–1.17]	0.02	1.05 [0.89–1.25]	0.55
Christian	0.73 [0.67–0.80]	< 0.001	0.97 [0.79–1.19]	0.768
Others	0.80 [0.71–0.90]	< 0.001	0.93 [0.74–1.17]	0.54
**MPCE**				
Rich	ref			
Middle	0.90 [0.85–0.96]	0.002	0.98 [0.86–1.12]	0.773
Poor	0.87 [0.82–0.92]	< 0.001	0.95 [0.85–1.06]	0.385
**Place of Residence**				
Rural	ref			
Urban	0.60 [0.57–0.63]	< 0.001	0.68 [0.61–0.76]	< 0.001
**Region**				
North	ref			
South	0.79 [0.73–0.85]	< 0.001	0.87 [0.75–1.02]	0.086
East	0.88 [0.81–0.95]	0.001	0.78 [0.66–0.92]	0.004
West	1.08 [1.00–1.17]	0.048	1.43 [1.21–1.69]	< 0.001
Central	1.57 [1.43–1.72]	< 0.001	2.14 [1.77–2.58]	< 0.001
Northeast	0.79 [0.73–0.86]	< 0.001	1.13 [0.93–1.38]	0.208
**Smoking**				
No	ref			
Yes	1.19 [1.13–1.25]	< 0.001	1.23 [1.11–1.37]	< 0.001
**Drinking**				
No	ref			
Yes	0.98 [0.92–1.05]	0.614	0.99 [0.87–1.13]	0.904
**Age at first job**				
Before 14	ref			
At or after 14	0.64 [0.60–0.69]	< 0.001	0.82 [0.73–0.93]	0.002
**Employment Status**				
Employed	ref			
Self-employed	1.32 [1.19–1.47]	< 0.001	1.07 [0.95–1.21]	0.252
**Current Main Job Quadrants**				
High Strain	ref			
Passive	0.95 [0.87–1.04]	0.293	0.84 [0.74–0.96]	0.007
Active	1.07 [1.00–1.14]	0.035	0.77 [0.64–0.92]	0.004
Low Strain	0.99 [0.92–1.08]	0.9	0.89 [0.80–1.01]	0.063
**Turnover Intention**				
Intention to Stay	ref			
Intention to leave	1.42 [1.21–1.67]	< 0.001	1.45 [1.18–1.79]	< 0.001
**ADL Limitation**				
No difficulty	ref			
Moderate	2.08 [1.94–2.23]	< 0.001	1.35 [1.15–1.58]	< 0.001
Severe	2.10 [1.89–2.33]	< 0.001	1.18 [0.87–1.59]	0.284
**IADL Limitation**				
No difficulty	ref			
Moderate	1.70 [1.60–1.81]	< 0.001	1.20 [1.06–1.36]	0.004
Severe	2.17 [2.03–2.31]	< 0.001	1.39 [1.18–1.63]	< 0.001
**Self-Rated Health (SRH)**				
Poor	ref			
Moderate	0.56 [0.53–0.60]	< 0.001	0.75 [0.66–0.85]	< 0.001
Good	0.26 [0.24–0.28]	< 0.001	0.42 [0.37–0.49]	< 0.001
**Life Satisfaction**				
Low	ref			
Medium	0.97 [0.91–1.03]	0.326	1.17 [1.03–1.32]	0.014
High	0.86 [0.81–0.91]	< 0.001	0.96 [0.85–1.07]	0.468
**Depressive Symptoms**				
No	ref			
Yes	2.52 [2.33–2.71]	< 0.001	1.28 [1.09–1.51]	0.002
**Insomnia**				
No	ref			
Yes	2.76 [2.62–2.90]	< 0.001	1.93 [1.73–2.15]	< 0.001
**Morbidity Status**				
No morbidity	ref			
Single morbidity	1.77 [1.67–1.87]	< 0.001	1.59 [1.42–1.78]	< 0.001
Multimorbidity	2.61 [2.46–2.77]	< 0.001	2.31 [2.02–2.65]	< 0.001
**Subjective Health Complaints (SHC)**				
No	ref			
Yes	2.26 [1.70–3.00]	< 0.001	2.04 [1.13–3.68]	0.018

Notes: Ref: Reference Category; OR: Odds Ratio; CI: Confidence Interval

## Data Availability

The LASI data used in this study are freely available in the public domain. The LASI was released through the websites of the Gateway to Global Ageing Data (https://g2aging.org) and the International Institute for Population Sciences (IIPS) (www.iipsindia.ac.in/lasi). A data request form is available on the IIPS website (https://iipsindia.ac.in/content/data-request). To access the data, users can register and submit a statement of purpose for the use of the data.

## Abbreviations

ADL: Activities of Daily Living
AP: Angina Pectoris
CHARLS: China Health and Retirement Longitudinal Study
HPA: Hypothalamic-Pituitary-Adrenal Axis
HRS: Health and Retirement Study
IADL: Instrumental Activities of Daily Living
ITL: Intention to Leave
ITS: Intention to Stay
JDC: Job-Demand Control
JSTAR: Japanese Study of Aging and Retirement
Korean: Longitudinal Study of Aging
LASI: Longitudinal Aging Study in India
MGNREGA: Mahatma Gandhi National Rural Employment Guarantee Act, 2005
MHAS: Mexican Health and Aging Study
MPCE: Monthly Per Capita Consumption Expenditure
NPSHEW: National Policy on Safety, Health and Environment at Workplace
NSS: National Sample Survey
OSH&WC: Occupational Safety, Health and Working Conditions Code
SHARE: Survey of Health, Aging and Retirement in Europe

## References

[1] Roth Gregory A., Mensah George A, Valentin. The Global Burden of Cardiovascular Diseases and Risks. 2020; 10.1016/j.jacc.2020.11.021 33309174

[2] World Health Organization. Cardiovascular diseases (CVDs). 2025.

[3] Kalra A, Jose AP, Prabhakaran P, Kumar A, Agrawal A, Roy A, The burgeoning cardiovascular disease epidemic in Indians – perspectives on contextual factors and potential solutions. Lancet Reg Health - Southeast Asia. 2023 Feb 10;12:100156. 37384064 10.1016/j.lansea.2023.100156 PMC10305862

[4] Ramesh S, Kosalram K. The burden of non-communicable diseases: A scoping review focus on the context of India. J Educ Health Promot. 2023 Feb;12(1). 10.4103/jehp.jehp_1113_22 PMC10127498 37113407

[5] Mohanty Sanjay K. Trend in out-of-pocket expenditure and catastrophic health spending in India, 2004–18. Mumbai: International Institute for Population Sciences (IIPS);

[6] Bloom David E, Cafiero-Fonseca E. T, Saxena, Adashi E. Economics of non-communicable diseases in India: the costs and returns on investment of interventions to promote healthy living and prevent, treat, and manage NCDs. Geneva: World Economic Forum; Harvard School of Public Health; 2014 p. 22–3.

[7] Angina - Symptoms and causes - Mayo Clinic.

[8] Belkić K, Schnall P, Landsbergis P, Baker D. The workplace and cardiovascular health: conclusions and thoughts for a future agenda. Occup Med Phila Pa. 2000;15(1):307–21, v–vi. 10702092

[9] Belkic KL, Landsbergis PA, Schnall PL, Baker D. Is job strain a major source of cardiovascular disease risk? Scand J Work Environ Health. 2004;30(2):85–128. 15127782 10.5271/sjweh.769

[10] Backé EM, Seidler A, Latza U, Rossnagel K, Schumann B. The role of psychosocial stress at work for the development of cardiovascular diseases: a systematic review. Int Arch Occup Environ Health. 2012 Jan;85(1):67–79. 21584721 10.1007/s00420-011-0643-6 PMC3249533

[11] Kuper H, Marmot M, Hemingway H. Systematic Review of Prospective Cohort Studies of Psychosocial Factors in the Etiology and Prognosis of Coronary Heart Disease. Semin Vasc Med. 2002;02(3):267–314. 10.1055/s-2002-35401 16222620

[12] Kivimki M, Virtanen M, Elovainio M, Kouvonen A, Vaananen A, Vahtera J. Work stress in the etiology of coronary heart disease?a meta-analysis. Scand J Work Environ Health. 2006;32(6):431–42. 17173200 10.5271/sjweh.1049

[13] Toivanen S. Social Determinants of Stroke as Related to Stress at Work among Working Women: A Literature Review. Stroke Res Treat. 2012;2012:1–10. 10.1155/2012/873678 PMC3518054 23251832

[14] Landsbergis P. Job Strain and Cardiovascular Disease. Annu Rev Public Health. 1994 Jan 1; 10.1146/annurev.pu.15.050194.002121 8054091

[15] Power N, Deschênes SS, Ferri F, Schmitz N. Job strain and the incidence of heart diseases: A prospective community study in Quebec, Canada. J Psychosom Res. 2020 Dec;139:110268. 33069052 10.1016/j.jpsychores.2020.110268

[16] Kivimaki M. Work stress and risk of cardiovascular mortality: prospective cohort study of industrial employees. BMJ [Internet]. 2002 Oct 19 [cited 2025 Sep 3];325(7369):857–857. Available from: https://www.bmj.com/lookup/doi/10.1136/bmj.325.7369.857 12386034 PMC129630

[17] Karasek R, Baker D, Marxer F, Ahlbom A, Theorell T. Job decision latitude, job demands, and cardiovascular disease: a prospective study of Swedish men. Am J Public Health. 1981 Jul;71(7):694–705. 7246835 10.2105/ajph.71.7.694 PMC1619770

[18] Panagiotakos DB, Chrysohoou C, Pitsavos C, Antoniou S, Vavouranakis E, Stravopodis P, The association between occupational stress and the risk of developing acute coronary syndromes: the CARDIO2000 Study. Cent Eur J Public Health. 2003 Mar;11(1):25–30. 12690800

[19] Kristensen TS, Hannerz H, Hugh A, Borg V. The Copenhagen Psychosocial Questionnaire-a tool for the assessment and improvement of the psychosocial work environment. Scand J Work Environ Health. 2005;31(6):438–49. 16425585 10.5271/sjweh.948

[20] Rosengren A, Hawken S, Ounpuu S, Sliwa K, Zubaid M, Almahmeed WA, Association of psychosocial risk factors with risk of acute myocardial infarction in 11119 cases and 13648 controls from 52 countries (the INTERHEART study): case-control study. Lancet Lond Engl. 2004 Sep 11;364(9438):953–62. 10.1016/S0140-6736(04)17019-0 15364186

[21] Steenland K, Johnson J, Nowlin S. A follow-up study of job strain and heart disease among males in the NHANES1 population. Am J Ind Med. 1997 Feb;31(2):256–60. 9028443 10.1002/(sici)1097-0274(199702)31:2<256::aid-ajim16>3.0.co;2-0

[22] Feveile HB, Olsen O, Burr FHM, Bach E. Danish work environment cohort study 2005: from idea to sampling design. Stat Transit - New Ser. 2007;8(3):441–58.

[23] Kivimäki M, Lawlor DA, Davey Smith G, Kouvonen A, Virtanen M, Elovainio M, Socioeconomic position, co-occurrence of behavior-related risk factors, and coronary heart disease: the Finnish Public Sector study. Am J Public Health. 2007 May;97(5):874–9. 17395837 10.2105/AJPH.2005.078691 PMC1854863

[24] Kivimaki M, Theorell T, Westerlund H, Vahtera J, Alfredsson L. Job strain and ischaemic disease: does the inclusion of older employees in the cohort dilute the association? The WOLF Stockholm Study. J Epidemiol Community Health. 2008 Apr 1;62(4):372–4. 18339833 10.1136/jech.2007.063578

[25] Kivimäki M, Nyberg ST, Batty GD, Fransson EI, Heikkilä K, Alfredsson L, Job strain as a risk factor for coronary heart disease: a collaborative meta-analysis of individual participant data. Lancet. 2012 Oct;380(9852):1491–7. 22981903 10.1016/S0140-6736(12)60994-5 PMC3486012

[26] Ferrario MM, Veronesi G, Bertù L, Grassi G, Cesana G. Job strain and the incidence of coronary heart diseases: does the association differ among occupational classes? A contribution from a pooled analysis of Northern Italian cohorts. BMJ Open. 2017 Jan 24;7(1):e014119. 10.1136/bmjopen-2016-014119 PMC5278242 28119392

[27] Slopen N, Glynn RJ, Buring JE, Lewis TT, Williams DR, Albert MA. Job strain, job insecurity, and incident cardiovascular disease in the Women’s Health Study: results from a 10-year prospective study. PloS One. 2012;7(7):e40512. 22815754 10.1371/journal.pone.0040512 PMC3399852

[28] MATTHEWS TA, CHEN L, LI J. Increased job strain and cardiovascular disease mortality: a prospective cohort study in U.S. workers. Ind Health. 2023 Jul;61(4):250–9. 35811129 10.2486/indhealth.2021-0233 PMC10398175

[29] Eaker ED, Sullivan LM, Kelly-Hayes M, D’Agostino RB, Benjamin EJ. Does job strain increase the risk for coronary heart disease or death in men and women? The Framingham Offspring Study. Am J Epidemiol. 2004 May 15;159(10):950–8. 15128607 10.1093/aje/kwh127

[30] Lee S, Colditz G, Berkman L, Kawachi I. A prospective study of job strain and coronary heart disease in US women. Int J Epidemiol. 2002 Dec;31(6):1147–53. 12540714 10.1093/ije/31.6.1147

[31] Wang C, Lê-Scherban F, Taylor J, Salmoirago-Blotcher E, Allison M, Gefen D, Associations of Job Strain, Stressful Life Events, and Social Strain With Coronary Heart Disease in the Women’s Health Initiative Observational Study. J Am Heart Assoc. 2021 Feb;10(5):e017780. 33618543 10.1161/JAHA.120.017780 PMC8174284

[32] Kivimäki M, Nyberg ST, Batty GD, Fransson EI, Heikkilä K, Alfredsson L, Job strain as a risk factor for coronary heart disease: a collaborative meta-analysis of individual participant data. The Lancet. 2012 Oct 27;380(9852):1491–7. 10.1016/S0140-6736(12)60994-5 PMC3486012 22981903

[33] Torén K, Schiöler L, Giang WK, Novak M, Söderberg M, Rosengren A. A longitudinal general population-based study of job strain and risk for coronary heart disease and stroke in Swedish men. BMJ Open. 2014 Mar 1;4(3):e004355. 10.1136/bmjopen-2013-004355 PMC3948640 24589825

[34] Padyab M, Blomstedt Y, Norberg M. No association found between cardiovascular mortality, and job demands and decision latitude: Experience from the Västerbotten Intervention Programme in Sweden. Soc Sci Med. 2014 Sep 1;117:58–66. 25047710 10.1016/j.socscimed.2014.07.033

[35] De Bacquer D, Pelfrene E, Clays E, Mak R, Moreau M, De Smet P, Perceived Job Stress and Incidence of Coronary Events: 3-Year Follow-up of the Belgian Job Stress Project Cohort. Am J Epidemiol. 2005 Mar 1;161(5):434–41. 15718479 10.1093/aje/kwi040

[36] Hlatky MA, Lam LC, Lee KL, Clapp-Channing NE, Williams RB, Pryor DB, Job Strain and the Prevalence and Outcome of Coronary Artery Disease. Circulation. 1995 Aug;92(3):327–33. 7634445 10.1161/01.cir.92.3.327

[37] Nyberg ST, Fransson EI, Heikkilä K, Alfredsson L, Casini A, Clays E, Job Strain and Cardiovascular Disease Risk Factors: Meta-Analysis of Individual-Participant Data from 47,000 Men and Women. PLOS ONE. 2013 Jun 20;8(6):e67323. 23840664 10.1371/journal.pone.0067323 PMC3688665

[38] Reed DM, Lacroix AZ, Karasek RA, Miller D, Maclean CA. OCCUPATIONAL STRAIN AND THE INCIDENCE OF CORONARY HEART DISEASE. Am J Epidemiol. 1989 Mar;129(3):495–502. 2916542 10.1093/oxfordjournals.aje.a115160

[39] Poli A, Catapano AL, Corsini A, Manzato E, Werba JP, Catena G, LDL-cholesterol control in the primary prevention of cardiovascular diseases: An expert opinion for clinicians and health professionals. Nutr Metab Cardiovasc Dis. 2023 Feb 1;33(2):245–57. 36566123 10.1016/j.numecd.2022.10.001

[40] Davalagi S, Amuje R, H S. Cardiovascular Risk Assessment Among People With Type 2 Diabetes Mellitus in Urban Slums of Central Karnataka, India. Cureus. 15(10):e46687. 37942395 10.7759/cureus.46687 PMC10629598

[41] Unnikrishnan AG, Sahay RK, Phadke U, Sharma SK, Shah P, Shukla R, Cardiovascular risk in newly diagnosed type 2 diabetes patients in India. PloS One. 2022;17(3):e0263619. 35358208 10.1371/journal.pone.0263619 PMC8970505

[42] Tharkar S, Viswanathan V. Effect of obesity on cardiovascular risk factors in urban population in South India. Heart Asia. 2010;2(1):145–9. 27325967 10.1136/ha.2009.000950 PMC4898498

[43] Shokeen D, Aeri BT. Prevalence of Cardio-metabolic Risk Factors: A Cross-sectional Study among Employed Adults in Urban Delhi, India. J Clin Diagn Res JCDR. 2017 Aug;11(8):LC01–4. 10.7860/JCDR/2017/29087.10336 PMC5620802 28969161

[44] Taqiuddin R, Ali MJ, Kimmatkar A, Lohana N, Anveshak, Siddiqui MM, Smoking is a predominant risk factor for coronary artery disease among Indians. Bioinformation. 2024 Jul 31;20(7):719–22. 39309573 10.6026/973206300200719 PMC11414331

[45] Zhang X, Sun Y, Ye S, Huang Q, Zheng R, Li Z, Associations between insomnia and cardiovascular diseases: a meta-review and meta-analysis of observational and Mendelian randomization studies. J Clin Sleep Med JCSM Off Publ Am Acad Sleep Med. 2024 Dec 1;20(12):1975–84. 10.5664/jcsm.11326 PMC11609828 39167428

[46] Halder P, Das S, Mamgai A, Nongkynrih B, Bishoi S, Chattopadhyay A, Association of sleep disorders with non-lab-based CVD risk score among Indian population aged above 45 years: insight from Longitudinal Aging Study in India. Int J Community Med Public Health. 2024 Jul 30;11(8):3113–21.

[47] Kokubo Y, Matsumoto C. Hypertension Is a Risk Factor for Several Types of Heart Disease: Review of Prospective Studies. Adv Exp Med Biol. 2017;956:419–26. 27815926 10.1007/5584_2016_99

[48] Biswas A, Singh SK, Singh RK. Linkages between Hypertension and Coronary Heart Disease in India: Evidence from India Human Development Survey-2 (2011–2012). Indian J Community Med Off Publ Indian Assoc Prev Soc Med. 2017;42(4):200–3. 10.4103/ijcm.IJCM_168_16 PMC5682717 29184318

[49] Chacko M, Sarma PS, Harikrishnan S, Zachariah G, Jeemon P. Family history of cardiovascular disease and risk of premature coronary heart disease: A matched case-control study. Wellcome Open Res. 2020 Jun 12;5:70. 32518841 10.12688/wellcomeopenres.15829.1 PMC7256470

[50] Jeemon P, Harikrishnan S, Ganapathi S, Sivasankaran S, Binukumar B, Padmanabhan S, Efficacy of a family-based cardiovascular risk reduction intervention in individuals with a family history of premature coronary heart disease in India (PROLIFIC): an open-label, single-centre, cluster randomised controlled trial. Lancet Glob Health. 2021 Oct;9(10):e1442–50. 34534488 10.1016/S2214-109X(21)00319-3

[51] Singh M, Singh S, Pandey MK, Singh S. Exploring the link between physical activity and cardiovascular disease among Indian elderly: Evidence from the Longitudinal Aging Study in India(LASI). Curr Probl Cardiol. 2024 Nov;49(11):102778. 39089412 10.1016/j.cpcardiol.2024.102778

[52] Portoghese I, Galletta M, Leiter MP, Finco G, d’Aloja E, Campagna M, Job Demand-Control-Support Latent Profiles and Their Relationships with Interpersonal Stressors, Job Burnout, and Intrinsic Work Motivation. Int J Environ Res Public Health. 2020 Dec 16;17(24). 10.3390/ijerph17249430 PMC7765581 33339208

[53] Karasek R. A. Jr. (1979). Job Demands, Job Decision Latitude, and Mental Strain Implications for Job Redesign. Administrative Science Quarterly, 24, 285–308. - References - Scientific Research Publishing.

[54] Burr H, Müller G, Rose U, Formazin M, Clausen T, Schulz A, The Demand–Control Model as a Predictor of Depressive Symptoms—Interaction and Differential Subscale Effects: Prospective Analyses of 2212 German Employees. Int J Environ Res Public Health. 2021 Aug 6;18(16):8328. 34444078 10.3390/ijerph18168328 PMC8391232

[55] Aboa-Eboulé C, Brisson C, Maunsell E, Mâsse B, Bourbonnais R, Vézina M, Job strain and risk of acute recurrent coronary heart disease events. JAMA. 2007 Oct 10;298(14):1652–60. 17925517 10.1001/jama.298.14.1652

[56] A Longitudinal Test of the Demand–Control Model Using Specific Job Demands and Specific Job Control | International Journal of Behavioral Medicine. 10.1007/s12529-010-9081-1 PMC2862948 20195810

[57] International Institute for Population Sciences (IIPS), National Programme for Health Care of Elderly (NPHCE), Ministry of Health and Family Welfare (MoHFW), Harvard T.H. Chan School of Public Health (HSPH), University of Southern California (USC). Longitudinal Ageing Study in India (LASI) Wave 1, 2017–18, Executive Summary. Mumbai: International Institute for Population Sciences; 2020 p. 17–8.

[58] International Institute for Population Sciences, Harvard T.H. Chan School of Public Health, University of Southern California. Longitudinal Ageing Study in India (LASI) Wave 1: Household, Individual, and Biomarker Questionnaires. Mumbai: International Institute for Population Sciences (IIPS); 2020.

[59] Rose GA. The diagnosis of ischaemic heart pain and intermittent claudication in field surveys. Bull World Health Organ. 1962;27(6):645–58. 13974778 PMC2555832

[60] Pengpid S, Peltzer K. Perceived discrimination and health outcomes among middle-aged and older adults in India: results of a national survey in 2017–2018. BMC Geriatr. 2021 Dec;21(1):559. 34663217 10.1186/s12877-021-02508-z PMC8522245

[61] Karasek R, Brisson C, Kawakami N, Houtman I, Bongers P, Amick B. The Job Content Questionnaire (JCQ): an instrument for internationally comparative assessments of psychosocial job characteristics. J Occup Health Psychol. 1998 Oct;3(4):322–55. 9805280 10.1037//1076-8998.3.4.322

[62] Jana A, Varghese JS, Naik G. Household air pollution and cognitive health among Indian older adults: Evidence from LASI. Environ Res. 2022 Nov 1;214:113880. 35820648 10.1016/j.envres.2022.113880

[63] Perceived discrimination and health outcomes among middle-aged and older adults in India: results of a national survey in 2017–2018 | BMC Geriatrics. 10.1186/s12877-021-02508-z PMC8522245 34663217

[64] Mahoney FI, Barthel DW. Functional evaluation: The Barthel Index: A simple index of independence useful in scoring improvement in the rehabilitation of the chronically ill. Md State Med J. 1965;14:61–5. 14258950

[65] VanderWeele TJ, Hawkley LC, Cacioppo JT. On the Reciprocal Association Between Loneliness and Subjective Well-being. Am J Epidemiol. 2012 Nov 1;176(9):777–84. 23077285 10.1093/aje/kws173 PMC3571255

[66] The CES-D Scale - Lenore Sawyer Radloff, 1977.

[67] Screening for Depression in the Older Adult: Criterion Validity of the 10-Item Center for Epidemiological Studies Depression Scale (CES-D) | Depressive Disorders | JAMA Internal Medicine | JAMA Network.

[68] A scale for the estimation of sleep problems in clinical research - ScienceDirect. 10.1016/0895-4356(88)90138-2 3351539

[69] The association between single and multiple chronic conditions and depression among older population in India: A comparative study between men and women. [cited 2025 Dec 17]; Available from: https://onlinelibrary.wiley.com/doi/10.1002/gps.5639 10.1002/gps.5639 34633709

[70] Cohort Profile: The Longitudinal Ageing Study in India (LASI) | International Journal of Epidemiology | Oxford Academic. 10.1093/ije/dyab266 PMC9365624 35021187

[71] Gore-Langton GR, Mansfield KE, Ravi P, Doubatty A, Alladi S, Kinra S, Infections and cognitive function, depression, and frailty: a cross-sectional study in the longitudinal aging study in India (LASI). BMC Public Health. 2025 Jul 2;25(1):2244. 40604674 10.1186/s12889-025-23490-w PMC12220408

[72] Ahmed W, Muhammad T, Maurya C, Akhtar SN. Prevalence and factors associated with undiagnosed and uncontrolled heart disease: A study based on self-reported chronic heart disease and symptombased angina pectoris among middle-aged and older Indian adults. PLOS ONE. 2023 Jun 28;18(6):e0287455. 37379277 10.1371/journal.pone.0287455 PMC10306230

[73] Billing E, Eriksson SV, Hjemdahl P, Rehnqvist N. Psychosocial variables in relation to various risk factors in patients with stable angina pectoris. J Intern Med. 2000;247(2):240–8. 10692087 10.1046/j.1365-2796.2000.00590.x

[74] Lin J, Yang D, Zhao X, Xie L, Xiong K, Hu L, The action logic of the older adults about health-seeking in South Rural China. BMC Public Health. 2023 Dec 12;23(1):2487. 38087231 10.1186/s12889-023-17314-y PMC10714459

[75] Townsend N, Timmis A, Erglis A, Miličić D, Perrone-Filardi P, Weidinger F, Challenges in the prevention, treatment and management of cardiovascular disease among older adults. Lancet Reg Health - Eur. 2025 Aug 21;56:101372. 41200013 10.1016/j.lanepe.2025.101372 PMC12587328

[76] Tawfik MY, Soliman HH, Abdel-Fatah ZF. Accuracy of self-perceived cardiovascular disease risk and factors predicting risk underestimation in perimenopausal and postmenopausal women in Ismailia, Egypt. J Egypt Public Health Assoc. 2024 Oct 1;99(1):24. 39349881 10.1186/s42506-024-00170-y PMC11442895

[77] Angina in Coronary Artery Disease Patients With and Without Diabetes: US National Health and Nutrition Examination Survey 2001–2010. 10.1002/clc.22488 PMC6490722 26694985

[78] Hemingway H, Langenberg C, Damant J, Frost C, Pyörälä K, Barrett-Connor E. Prevalence of angina in women versus men: a systematic review and meta-analysis of international variations across 31 countries. Circulation. 2008 Mar 25;117(12):1526–36. 18347213 10.1161/CIRCULATIONAHA.107.720953

[79] Nazareth I, D’Costa G, Kalaitzaki E, Vaidya R, King M. Angina in primary care in Goa, India: sex differences and associated risk factors. Heart Asia. 2010;2(1):28–35. 27325939 10.1136/ha.2009.001255 PMC4898508

[80] Prevalence of cardiovascular morbidities in Myanmar | BMC Research Notes | Full Text. 10.1186/s13104-017-2422-2 PMC5312437 28202069

[81] Reddy KS, Prabhakaran D, Jeemon P, Thankappan KR, Joshi P, Chaturvedi V, Educational status and cardiovascular risk profile in Indians. Proc Natl Acad Sci. 2007 Oct 9;104(41):16263–8. 17923677 10.1073/pnas.0700933104 PMC2042195

[82] Educational Attainment and Lifetime Risk of Cardiovascular Disease | Cardiology | JAMA Cardiology | JAMA Network. 10.1001/jamacardio.2023.3990 PMC10620672 37910110

[83] Tao J, Zhao X, Li B, Sun H, Hu Y, Chen S, Associations of educational attainment and traditional risk factor control with cardiovascular disease. Am J Prev Cardiol. 2025 Sep;23:101031. 40585338 10.1016/j.ajpc.2025.101031 PMC12206016

[84] Liu W, Lin Q, Fan Z, Cui J, Wu Y. Education and cardiovascular diseases: a Mendelian randomization study. Front Cardiovasc Med. 2024 Feb 15;11:1320205. 38426117 10.3389/fcvm.2024.1320205 PMC10902100

[85] Vellakkal S, Subramanian SV, Millett C, Basu S, Stuckler D, Ebrahim S. Socioeconomic inequalities in non-communicable diseases prevalence in India: disparities between self-reported diagnoses and standardized measures. PloS One. 2013;8(7):e68219. 23869213 10.1371/journal.pone.0068219 PMC3712012

[86] Patel S, Ram F, Patel SK, Kumar K. Association of behavioral risk factors with self-reported and symptom or measured chronic diseases among adult population (18–69 years) in India: evidence from SAGE study. BMC Public Health. 2019 May 14;19(1):560. 31088447 10.1186/s12889-019-6953-4 PMC6518500

[87] Ahmed W, Dixit P. Effect of chronic lung diseases on angina pectoris among Indian adults: longitudinal ageing study in India. Sci Rep. 2024 Jan 1;14(1):2372. 38287095 10.1038/s41598-024-52786-x PMC10825144

[88] Ke C, Gupta R, Xavier D, Prabhakaran D, Mathur P, Kalkonde YV, Divergent trends in ischaemic heart disease and stroke mortality in India from 2000 to 2015: a nationally representative mortality study. Lancet Glob Health. 2018 Aug 1;6(8):e914–23. 30012272 10.1016/S2214-109X(18)30242-0 PMC6942542

[89] Zaman MJS, Loret de Mola C, Gilman RH, Smeeth L, Miranda JJ. The prevalence of angina symptoms and association with cardiovascular risk factors, among rural, urban and rural to urban migrant populations in Peru. BMC Cardiovasc Disord. 2010 Oct 8;10:50. 20932298 10.1186/1471-2261-10-50 PMC2964551

[90] Geldsetzer P, Manne-Goehler J, Theilmann M, Davies JI, Awasthi A, Danaei G, Geographic and sociodemographic variation of cardiovascular disease risk in India: A cross-sectional study of 797,540 adults. PLoS Med. 2018 Jun;15(6):e1002581. 29920517 10.1371/journal.pmed.1002581 PMC6007838

[91] Gupta R, Guptha S, Sharma KK, Gupta A, Deedwania P. Regional variations in cardiovascular risk factors in India: India heart watch. World J Cardiol. 2012 Apr 26;4(4):112–20. 22558490 10.4330/wjc.v4.i4.112 PMC3342579

[92] Prabhakaran D, Jeemon P, Sharma M, Roth GA, Johnson C, Harikrishnan S, The changing patterns of cardiovascular diseases and their risk factors in the states of India: the Global Burden of Disease Study 1990–2016. Lancet Glob Health. 2018 Dec 1;6(12):e1339–51. 30219317 10.1016/S2214-109X(18)30407-8 PMC6227386

[93] Buchanan DM, Arnold SV, Gosch KL, Jones PG, Longmore LS, Spertus JA, Association of Smoking Status With Angina and Health-Related Quality of Life After Acute Myocardial Infarction. Circ Cardiovasc Qual Outcomes. 2015 Sep;8(5):493–500. 26307130 10.1161/CIRCOUTCOMES.114.001545 PMC4703446

[94] Quashie NT, D’Este C, Agrawal S, Naidoo N, Kowal P. Prevalence of angina and co-morbid conditions among older adults in six low- and middle-income countries: Evidence from SAGE Wave 1. Int J Cardiol. 2019 Jun 15;285:140–6. 30879938 10.1016/j.ijcard.2019.02.068

[95] Chaar S, Yoon J, Abdulkarim J, Villalobos J, Garcia J, López Castillo H. Angina Outcomes in Secondhand Smokers: Results from the National Health and Nutrition Examination Survey 2007–2018. Avicenna J Med. 2022 Jul 3;12(2):73–80. 35833157 10.1055/s-0042-1750730 PMC9272456

[96] Merry AHH, Boer JMA, Schouten LJ, Feskens EJM, Verschuren WMM, Gorgels APM, Smoking, alcohol consumption, physical activity, and family history and the risks of acute myocardial infarction and unstable angina pectoris: a prospective cohort study. BMC Cardiovasc Disord. 2011 Mar 24;11:13. 21435252 10.1186/1471-2261-11-13 PMC3073941

[97] Williams EC, Bryson CL, Sun H, Au DH, Bradley KA. Association between Alcohol Use and Angina Symptoms among Outpatients from the Veterans Health Administration. J Addict Med. 2018;12(2):143–9. 29334512 10.1097/ADM.0000000000000379 PMC5847476

[98] Ding C, O’Neill D, Bell S, Stamatakis E, Britton A. Association of alcohol consumption with morbidity and mortality in patients with cardiovascular disease: original data and meta-analysis of 48,423 men and women. BMC Med. 2021 Jul 27;19(1):167. 34311738 10.1186/s12916-021-02040-2 PMC8314518

[99] Zhang Z, Zeng C, Chen Z, Liu P, Gao J, Guo Q, Age at job initiation and risk of coronary heart disease: findings from the UK biobank cohort study. BMC Public Health. 2023 Oct 30;23(1):2123. 37899473 10.1186/s12889-023-17034-3 PMC10614325

[100] Tsai CC, Chuang SY, Hsieh IC, Ho LH, Chu PH, Jeng C. The association between psychological distress and angina pectoris: A population-based study. PloS One. 2019;14(11):e0224451. 31703084 10.1371/journal.pone.0224451 PMC6839898

[101] Sacker A, Bartley MJ, Frith D, Fitzpatrick RM, Marmot MG. The relationship between job strain and coronary heart disease: evidence from an English sample of the working male population. Psychol Med. 2001 Feb;31(2):279–90. 11232915 10.1017/s0033291701003270

[102] Netterstrøm B, Kristensen TS, Møller L, Jensen G, Schnohr P. Angina pectoris, job strain, and social status: A cross-sectional study of employed urban citizens. Int J Behav Med. 1998 Dec 1;5(4):312–22. 16250698 10.1007/BF03003882

[103] Xu S, Huang Y, Xiao J, Zhu W, Wang L, Tang H, The association between job strain and coronary heart disease: A meta-analysis of prospective cohort studies. Ann Med. 2015 Aug 18;47(6):512–8. 26416502 10.3109/07853890.2015.1075658

[104] Lallukka T, Chandola T, Hemingway H, Marmot M, Lahelma E, Rahkonen O. Job strain and symptoms of angina pectoris among British and Finnish middle-aged employees. J Epidemiol Community Health. 2009 Dec;63(12):980–5. 19473995 10.1136/jech.2008.085878

[105] Popovic D, Lavie CJ. Stress, Cardiovascular Diseases and Exercise – A Narrative Review. Heart Mind. 2023 Mar;7(1):18.

[106] Black PH, Garbutt LD. Stress, inflammation and cardiovascular disease. J Psychosom Res. 2002 Jan;52(1):1–23. 11801260 10.1016/s0022-3999(01)00302-6

[107] Henein MY, Vancheri S, Longo G, Vancheri F. The Impact of Mental Stress on Cardiovascular Health—Part II. J Clin Med. 2022 Jul 28;11(15). 10.3390/jcm11154405 PMC9369438 35956022

[108] Acute mental stress drives vascular inflammation and promotes plaque destabilization in mouse atherosclerosis | European Heart Journal | Oxford Academic. 10.1093/eurheartj/ehab371 PMC8516477 34279021

[109] Evans E, Jacobs M, Fuller D, Hegland K, Ellis C. Allostatic Load and Cardiovascular Disease: A Systematic Review. Am J Prev Med. 2025 Jun;68(6):1072–9. 40054704 10.1016/j.amepre.2025.02.016 PMC13135696

[110] Sara JD, Prasad M, Eleid MF, Zhang M, Widmer RJ, Lerman A. Association Between Work-Related Stress and Coronary Heart Disease: A Review of Prospective Studies Through the Job Strain, Effort-Reward Balance, and Organizational Justice Models. J Am Heart Assoc. 2018 May;7(9):e008073. 29703810 10.1161/JAHA.117.008073 PMC6015274

[111] Occupational stress and the risk of turnover: a large prospective cohort study of employees in Japan | BMC Public Health. 10.1186/s12889-020-8289-5 PMC7001282 32019535

[112] Nakajima H, Yoshioka J, Totsuka N, Miyazawa I, Usui T, Urasawa N, Activities of daily living as an additional predictor of complications and outcomes in elderly patients with acute myocardial infarction. Clin Interv Aging. 2016 Aug 24;11:1141–7. 27601890 10.2147/CIA.S107136 PMC5003512

[113] Hu Z, Zheng B, Kaminga AC, Zhou F, Xu H. Association Between Functional Limitations and Incident Cardiovascular Diseases and All-Cause Mortality Among the Middle-Aged and Older Adults in China: A Population-Based Prospective Cohort Study. Front Public Health. 2022 Feb 11;10:751985. 35223720 10.3389/fpubh.2022.751985 PMC8873112

[114] Roy AR, Killian JM, Schulte PJ, Roger VL, Dunlay SM. Activities of Daily Living and Outcomes in Patients with Advanced Heart Failure. Am J Med. 2022 Dec 1;135(12):1497–1504.e2. 36063861 10.1016/j.amjmed.2022.08.009 PMC9691584

[115] Kazibwe R, Muhammad AI, Singleton MJ, Evans JK, Chevli PA, Namutebi JH, Self-rated health and risk of incident cardiovascular events among individuals with hypertension. J Hypertens. 2024 Sep 1;42(9):1573–80. 39088765 10.1097/HJH.0000000000003762 PMC11294676

[116] Mavaddat N, Parker RA, Sanderson S, Mant J, Kinmonth AL. Relationship of Self-Rated Health with Fatal and Non-Fatal Outcomes in Cardiovascular Disease: A Systematic Review and Meta-Analysis. PLOS ONE. 2014 Jul 30;9(7):e103509. 25076041 10.1371/journal.pone.0103509 PMC4116199

[117] Holm AE, Gomes LC, Wegener A, Lima KO, Matos LO, Vieira IVM, Is self-rated health associated with cardiovascular risk factors and disease in a low-income setting? A cross-sectional study from the Amazon Basin of Brazil. BMJ Open. 2022 Aug 30;12(8):e058277. 10.1136/bmjopen-2021-058277 PMC9438027 36041756

[118] Newman AB, Enright PL, Manolio TA, Haponik EF, Wahl PW, Group O behalf of the CHSR. Sleep Disturbance, Psychosocial Correlates, and Cardiovascular Disease in 5201 Older Adults: The Cardiovascular Health Study. J Am Geriatr Soc. 1997;45(1):1–7. 8994480 10.1111/j.1532-5415.1997.tb00970.x

[119] Zeng C, Ke Y, Li H, Zhang C, Chen J, Chen M. Causal Effects of Sleep Traits on Angina Pectoris: Mediation by Cardiovascular Risk Factors. Nat Sci Sleep. 2025;17:297–311. 39959816 10.2147/NSS.S484582 PMC11829606

[120] Sofi F, Cesari F, Casini A, Macchi C, Abbate R, Gensini GF. Insomnia and risk of cardiovascular disease: a meta-analysis. Eur J Prev Cardiol. 2014 Jan;21(1):57–64. 22942213 10.1177/2047487312460020

[121] Asplund R. Sleep and cardiac diseases amongst elderly people. J Intern Med. 1994 Jul;236(1):65–71. 8021575 10.1111/j.1365-2796.1994.tb01121.x

[122] Zheng W, Huang X, Wang X, Suo M, Yan Y, Gong W, Impact of multimorbidity patterns on outcomes and treatment in patients with coronary artery disease. 10.1093/ehjopen/oeae009 PMC10970685 38544919

[123] Violán C, Bejarano-Rivera N, Foguet-Boreu Q, Roso Llorach A, Pons-Vigués M, Martin Mateo M, The burden of cardiovascular morbidity in a European Mediterranean population with multimorbidity: a cross-sectional study. BMC Fam Pract. 2016 Nov 3;17(1):150. 27809772 10.1186/s12875-016-0546-4 PMC5093992

[124] Clifford C, Löwe B, Kohlmann S. Characteristics and predictors of persistent somatic symptoms in patients with cardiac disease. Sci Rep. 2024 Oct 26;14(1):25517. 39462010 10.1038/s41598-024-76554-z PMC11513025

[125] Kohlmann S, Gierk B, Hümmelgen M, Blankenberg S, Löwe B. Somatic Symptoms in Patients With Coronary Heart Disease: Prevalence, Risk Factors, and Quality of Life. JAMA Intern Med. 2013 Aug 12;173(15):1469–71. 23780333 10.1001/jamainternmed.2013.6835

[126] Nabi H, Hall M, Koskenvuo M, Singh-Manoux A, Oksanen T, Suominen S, Psychological and somatic symptoms of anxiety and risk of coronary heart disease: the health and social support prospective cohort study. Biol Psychiatry. 2010 Feb 15;67(4):378–85. 19819425 10.1016/j.biopsych.2009.07.040 PMC2963017

